# Verbal and non-verbal communication skills including empathy during history taking of undergraduate medical students

**DOI:** 10.1186/s12909-018-1260-9

**Published:** 2018-07-03

**Authors:** Daniela Vogel, Marco Meyer, Sigrid Harendza

**Affiliations:** Department of Internal Medicine, University Medical Center Hamburg-Eppendorf, Universitätsklinikum Hamburg-Eppendorf III. Medizinische Klinik Martinistr. 52, D-20246 Hamburg, Germany

**Keywords:** CARE, Communications skills, Empathy, History taking, Undergraduate medical education

## Abstract

**Background:**

Verbal and non-verbal aspects of communication as well as empathy are known to have an important impact on the medical encounter. The aim of the study was to analyze how well final year undergraduate medical students use skills of verbal and non-verbal communication during history-taking and whether these aspects of communication correlate with empathy and gender.

**Methods:**

During a three steps performance assessment simulating the first day of a resident 30 medical final year students took histories of five simulated patients resulting in 150 videos of physician-patient encounters. These videos were analyzed by external rating with a newly developed observation scale for the verbal and non-verbal communication and with the validated CARE-questionnaire for empathy. One-way ANOVA, t-tests and bivariate correlations were used for statistical analyses.

**Results:**

Female students showed signicantly higher scores for verbal communication in the case of a female patient with abdominal pain (*p* < 0.05), while male students started the conversations significantly more often with an open question (*p* < 0.05) and interrupted the patients significantly later in two cases than female students (*p* < 0.05). The number of W-questions asked by all students was significantly higher in the case of the female patient with abdominal pain (*p* < 0.05) and this patient was interrupted after the beginning of the interview significantly earlier than the patients in the other four cases (*p* < 0.001). Female students reached significantly higher scores for non-verbal communication in two cases (*p* < 0.05) and showed significantly more empathy than male students in the case of the female patient with abdominal pain (*p* < 0.05). In general, non-verbal communication correlated significantly with verbal communication and with empathy while verbal communication showed no significant correlation with empathy.

**Conclusions:**

Undergraduate medical students display differentiated communication behaviour with respect to verbal and non-verbal aspects of communication and empathy in a performance assessment and special differences could be detected between male and female students. These results suggest that explicit communication training and feedback might be necessary to raise students’ awareness for the different aspects of communication and their interaction.

## Background

Verbal as well as non-verbal communication and empathy play an important role in patient-physician encounters. Affiliative styles of communication were shown to be positively related to patients’ satisfaction with a physician while a negative association of patients’ satisfaction with a physician correlated with dominant/active communication styles [1, 2]. An affiliative style of communication reduced patients’ anxiety and facilitated their openness whereas a dominant/active communication style displayed reprimanding or condescending features, which resulted in reduced patient disclosure and compliance [1]. A physician’s communication style seems to be very important for the first encounter with a new patient, because patients build their first impression of a physician by a strong focus on his or her communication style [1]. Furthermore, patients’ outcomes are congruently associated with their feelings about aspects of communication after the consultation with a physician [3–5]. In patient-physician communication, a patient-centred approach is crucial which includes five aspects: a biopsychosocial perspective, the ‘patient-as-person’, sharing power and responsibility, therapeutic alliance, and ‘doctor-as-person’ [6].

Communication in medical encounters comprises verbal and non-verbal aspects. If these forms of communication are inconsistent or contradictory, the non-verbal messages tend to override the verbal messages [7]. Mehrabian and Ferris even developed a formula for verbal and non-verbal effects of a message: total impact = .07 verbal + .38 vocal + .55 facial [8]. For patient-physician encounters, important non-verbal signs by a physician, which influence a patient’s disclosure of history details in a consultation are eye contact, posture, the tone of voice, head nods, gesture, and the postural position [9–12]. Relationships could be detected between some of these non-verbal signs, patients’ satisfaction [13, 14], physicians’ workload [15], physicians’ malpractice claim history [10], patients’ recall of medical information, and compliance with keeping appointments and medical regimens [7, 16, 17]. Furthermore, the position of the patients facing forward to the physician in a 45-degree angle was the best regarding the frequency of eye contact [18]. Several studies reported a correlation between using records like computer or paper and the loss of eye contact while making notes. This lead to a reduced frequency of asking about psychosocial aspects in a patient’s medical history, a reduced response to emotional aspects provided by the patients, and to a reduced disclosure of history details by the patients [19–23].

Physicians who express empathy in patient encounters by acting in a warm, friendly and reassuring way seem to be more effective in reaching patients’ satisfaction and recovering [24]. Empathy is of great significance for better healthcare outcomes as part of a warm and friendly communication style [25–28]. Communication trainings are an effective teaching method to improve technical communication skills as well as empathy as a communication skill [29, 30]. However, the focus of communication trainings for undergraduate medical students is often on particular aspects of communication, e.g. informed consent or breaking bad news [31, 32]. Whether medical students are able to pay attention to all aspects of adequate and patient-centred communication in complex situations they will encounter in their future workplace is not known. Furthermore, gender has been reported to have an effect on patient-physician communication. Female physicians showed greater engagement in patient-centered communication and their consultation times were longer [33, 34]. On the Jefferson Scale of Physician Empathy, female medical students scored significantly higher than male medical students [35]. The aim of our study was to analyze, whether and how well final year undergraduate medical students use skills of verbal and non-verbal communication during history taking and whether a correlation can be found with the empathy shown towards a standardized patient as observed by an external rater and with gender.

## Methods

Until 2012, the undergraduate medical curriculum at the medical faculty of Hamburg consisted of two pre-clinical years, three clinical years, and a sixths practice year [36]. During the two pre-clinical years, history taking is taught in seminars with a focus on verbal communication skills and history taking techniques. In the three clinical years, which were organized in six thematic blocks, verbal and nonverbal history taking skills are practiced in bedside teaching courses on the hospital wards in the different medical disciplines. Seminars with standardized patients were scheduled in the thematic block “psycho-social medicine”, with a particular focus on empathetic communication including feedback by the actors.

In July 2011, 30 undergraduate medical students near graduation from the medical faculty of Hamburg University participated in a performance assessment resembling the first day of a beginning resident in hospital called UHTRUST (Utrecht Hamburg Trainee Responsibility for Unfamiliar Situations Test), which had been developed in a cooperation between the universities of Utrecht and Hamburg [37]. This assessment consisted per student of five 10-min consultations for history taking with standardized patients, followed by 3 hours where participants could gather further information and also interacted with nurses and other staff, and ended with a report to the individual supervisor about the five patients (30 min). All 150 patient interviews were videotaped and the content of the patient cases is described in further details elsewhere [38, 39]. In brief, the contents for the five different cases are: Case 1: coeliac disease (the mother of a 5-year-old girl describing the girls fatigue and abdominal pain), case 2: granulomatous polyangiitis (a 53-year-old missionary from Africa visiting his sister in Germany, complaining of hemoptysis and weakness), case 3: perforated sigmoid diverticulitis (a 58-year-old woman presenting with abdominal pain), case 4: myasthenia gravis (a 65-year-old female with difficulties to speak and to swallow who is accompanied by her husband), case 5: varicella zoster infection (a 36-year-old male under immunosuppressive therapy for rheumatoid arthritis and complaining of fever). The medical scenarios were developed by medical experts from the universities of Utrecht and Hamburg based on certain facets of competences as described earlier [37].

For the observation of the videos, three different instruments were used. Empathy was rated with the German version of the so-called CARE (Consultation and Relational Empathy) questionnaire [40]. The questionnaire was developed originally for assessment of physicians’ empathy by patients and contains 10 items, which have to be rated on a 5-point Likert scale (1: “I totally disagree” to 5: “I totally agree”). We used it in our study for external rating of empathy with only eight items because two items refer to therapy, which is not applicable in our setting of mere history taking. For verbal communication, six aspects have been adapted from the literature and were combined in a newly designed observation form: “uses suitable language” [41], “keeps the conversation running” [42], and “summarizes what has been said” [42], which were rated on a 3-point Likert scale (0: “does not apply”, 1: “applies partly”, 2: “applies fully”). For these aspects, a maximum score of 6 could be reached per patient case. Two further aspects, “opens the conversation with an open question” and “closes the conversation with an open question” were adapted from Sennekamp et al. [43] and answered dichotomously. Furthermore, the number of W-questions (what, when, why etc.) was counted per patient interview. For nonverbal communication, five aspects were combined in a new observation form: adequate body posture, appropriate facial expressions, eye contact, and appropriate tone of voice [18, 42, 43] were rated on a 3-point Likert scale (0: “not shown”, 1: “partially shown”, 2: “completely shown”). If all components were complete shown, a maximum value of 8 points per scenario could be reached. Additionally, the time between the end of the first question of the participant to the first interruption during the patient’s answer was measured.

All rating forms were piloted. Two raters rated 15 patient interviews (in each case five interviews of three patients) independently. The limit of acceptable difference was defined in the following way: two points for the non-verbal form and one point for the verbal form. Difference in agreements were 1.5 for the non-verbal and 1.2 for the verbal form. Hence, no further revision was necessary. The CARE questionnaire was piloted with ten patient interviews (five interviews of two patients) by two independent raters (MM, a physician, and DV, an educationalist). A maximum difference of eight points for the total score was defined as acceptable. After repeated discussion of the rating aspects, an acceptable agreement was reached. The videos were watched once for each questionnaire, i.e. three times in total. One-way ANOVA as well as t-tests and bivariate correlations were used for statistical analyses.

## Results

### Sample

Of the 30 participating final year students, 22 were female and eight were male. This resulted in 150 patient interviews altogether with 110 patient histories taken by female students and 40 histories taken by male students. Fifty percent of the students were between 24 and 25 years old, 46.7% were between 26 and 30 years old, and one student was 36 years old. All students were in the final year of their undergraduate medical curriculum lasting 6 years in total.

All students showed the highest verbal competence in case 4 (woman with difficulties to speak and swallow, accompanied by her husband) (Table 1). Female students were rated significant higher for their verbal communication over all cases (*p* < 0.05) and particularly in case 3 (woman with abdominal pain) than male students (*p* < 0.05). All students asked the highest number of W-questions in case 3 (Table 2). This number was significantly different versus case 2 (*p* < 0.05) and case 4 (*p* < 0.01). Significant gender differences could not be found.

**Table 1:** Verbal communication

Case	Total (*n* = 30)	Female (*n* = 22)	Male (*n* = 8)
	M ± SD	M ± SD	M ± SD
1	3.73 ± 1.12	3.86 ± 1.20	3.38 ± 1.06
2	3.67 ± 1.30	3.82 ± 1.18	3.25 ± 1.58
3	3.80 ± 1.30	**4.14 ± 1.13** ^**a**^	2.88 ± 1.36
4	4.10 ± 1.27	4.23 ± 1.11	3.75 ± 1.67
5	3.94 ± 0.98	4.09 ± 0.87	3.50 ± 1.20
Total	3.84 ± 1.20	**4.03 ± 1.10** ^**a**^	3.35 ± 1.35

^a^*p* < 0.05

The bold numbers in the tables are significant

**Table 2:** Average number of W-questions

Case	Total (*n* = 30)	Female (*n* = 22)	Male (*n* = 8)
	M ± SD	M ± SD	M ± SD
1	4.57 ± 2.33	4.36 ± 2.10	5.13 ± 3.14
2	4.37 ± 2.82	4.09 ± 2.65	5.13 ± 3.31
3	**6.03 ± 2.83** ^**a, b**^	6.09 ± 2.51	5.88 ± 5.80
4	4.10 ± 2.20	4.36 ± 2.28	3.38 ± 1.92
5	5.20 ± 2.46	5.55 ± 2.46	4.25 ± 2.32
Total	4.85 ± 2.60	4.89 ± 2.48	4.75 ± 2.95

^a^Total, case 3 vs. 2 (*p* < 0.05), ^b^ Total, case 3 vs. 4 (*p* < 0.01)

The bold numbers in the tables are significant

Students interrupted the patient in case 3, compared to all other cases, significantly earlier, already after 7.5 ± 6.4 s, while they interrupted the patient in case 2 latest after 32.7 ± 22.0 s (Table 3). Male students interrupted the patients over all cases significantly later than female students (*p* < 0.05), particularly in case 1 (*p* < 0.01), case 2 (*p* < 0.05), and case 5 (*p* < 0.05). About 65% of the students started the interview with an open question, 87.5% of the male and 56.4% of the female students, which shows a significant gender difference of *p* < 0.05. This difference is also found for the first, fourth and fifth case (*p* < 0.01; *p* < 0.01; *p* < 0.05). The interview of case 5 was started significantly more frequently with an open question than the interview of the second case (*p* < 0.05). Only one third of the students closed the interview with an open question (32.7% of the female students versus 30.0% of the male students). The interview of case 1 was closed significantly more frequently with an open question (46.7%) than the interview of case 4 (23.3%, *p* < 0.05). With respect to non-verbal communication (Table 4), female students displayed significantly more signs of non-verbal communication over all cases (*p* < 0.01), particularly in case 3 (*p* < 0.05) and 4 (*p* < 0.01) than male students did.

**Table 3:** Time to first interruption of the patient in seconds

Case	Total (*n* = 30)	Female (*n* = 22)	Male (*n* = 8)
	M ± SD	M ± SD	M ± SD
1	20.7 ± 14.9	17.2 ± 14.8	**30.4 ± 11.1** ^**b**^
2	32.7 ± 22.0	29.3 ± 20.0	**41.9 ± 25.9** ^**a**^
3	**7.5 ± 6.4** ^**c**^	7.6 ± 7.0	7.5 ± 4.8
4	31.7 ± 17.5	29.1 ± 19.0	38.6 ± 10.6
5	25.7 ± 15.9	22.8 ± 14.0	**33.5 ± 19.0** ^**a**^
Total	23.7 ± 18.4	21.2 ± 17.4	**30.4 ± 19.6** ^**a**^

^a^*p* < 0.05, ^b^*p* < 0.01, ^c^*p* < 0.001

The bold numbers in the tables are significant

**Table 4:** Non-verbal communication

Case	Total (*n* = 30)	Female (*n* = 22)	Male (*n* = 8)
	M ± SD	M ± SD	M ± SD
1	4.73 ± 1.34	4.73 ± 1.35	4.75 ± 1.39
2	5.07 ± 1.57	5.36 ± 1.47	4.25 ± 1.67
3	4.63 ± 1.71	**5.00 ± 1.51** ^**a**^	3.63 ± 1.92
4	5.10 ± 1.87	**5.64 ± 1.74** ^**b**^	3.63 ± 1.40
5	4.73 ± 1.46	4.77 ± 1.63	4.63 ± 0.92
Total	4.85 ± 1.59	**5.10 ± 1.59** ^**b**^	4.18 ± 1.50

^a^*p* < 0.05, ^b^*p* < 0.01

The bold numbers in the tables are significant

With respect to empathy, no differences were found for all participants between the five cases (Table 5). For case 3, female students were rated by an external rater to be more empathetic than male students (*p* < 0.05). Overall verbal communication correlated significantly with non-verbal communication (*p* < 0.01; *r* = .524) but not with empathy. Empathy correlated significantly with non-verbal communication (*p* < 0.01; *r* = .371).

**Table 5:** Empathy evaluated with the CARE questionnaire

Case	Total (*n* = 30)	Female (*n* = 22)	Male (*n* = 8)
	M ± SD	M ± SD	M ± SD
1	28.7 ± 4.1	28.8 ± 4.0	28.5 ± 4.7
2	28.2 ± 5.5	28.6 ± 6.2	27.1 ± 3.4
3	28.5 ± 4.1	**29.6 ± 4.1** ^**a**^	25.4 ± 2.1
4	27.7 ± 4.5	28.0 ± 4.8	26.9 ± 3.7
5	27.2 ± 4.4	27.3 ± 4.2	26.9 ± 5.1
Total	28.1 ± 4.5	28.5 ± 4.7	26.9 ± 3.9

^a^*p* < 0.05

The bold numbers in the tables are significant

## Discussion

The objective of the study was to analyze how well final year undergraduate medical students use skills of verbal and non-verbal communication during history-taking and whether these aspects of communication correlate with empathy. We found a significant correlation between verbal and non-verbal communication in our study. This could be interpreted as a sign for congruent communication, which is important for the interpersonal relationship [44]. This study also showed that inconsistent messages were associated with greater interpersonal distances, which might hamper the patient-physician relationship. The significant correlation of empathy with non-verbal communication but not with verbal communication supports the finding that physician involvement was associated with higher patient ratings of empathy and satisfaction [45]. Gaze and body orientation, two aspects of non-verbal communication, which were part of our observation scale, have been demonstrated to be important links to the perception of clinical empathy [46]. Furthermore, our findings support the idea, that non-verbal behaviour might be more important than verbal messages in the communication of empathy [47] and serves as the primary vehicle for expressing emotions [45].

Participants reached the highest scores for verbal and non-verbal communication skills with case 4, the female patient with difficulties to speak and swallow whom her husband accompanied. The fact that the patient’s speech was slurred and that a relative accompanied her might have drawn the students’ attention to particularly careful communication. From patients with aphasia it is known, that family members want physicians to try to communicate with the patient [48]. Whether students behaved in this manner instinctively or whether they were encouraged to behave in this way by training cannot be distinguished. With respect to gender differences, female students reached significantly higher scores than male students for verbal and non-verbal communication skills over all cases and in case 3, the woman with abdominal pain, and they received significantly higher scores for empathy in case 3. For communicating error disclosures, it is known that female physicians smiled more and were more attentive than male physicians were [49]. This might also be the case in our patient scenario with a female patient who was brought to the consulting room in a wheelchair because of severe abdominal pain. Another study reports empirical evidence for more signs of non-verbal and verbal ways of communication in female physicians including smiling, disclosing information about themselves, and encouraging and facilitating others to talk more freely [50]. The higher ratings for empathy are in line with another study, which showed that female students were more patient-centred than male students [51]. Furthermore, students in this study were more attuned to the concerns of patients of their own gender [51], which also might be the case with the patient in case 3.

The patient in case 3 was interrupted most frequently after the shortest interval from the start of the conversation and the highest number of W-questions was asked. Furthermore, in case 3 students have been shown to have asked significantly more questions about medical details than in any other case [38]. Case 3 covers the symptom abdominal pain, which is taught repeatedly in our 6-year undergraduate medical curriculum [36] and W-questions are important to distinguish differential diagnoses [52]. Our results might demonstrate, that students have studied the workup of patients with abdominal pain well. However, female students were found to interrupt patients significantly earlier than male students over all cases. With respect to interrupting a conversation, the important finding in the literature is that the quality of the interruption needs to be distinguished as there is a cooperative and an intrusive way of interrupting [53]. In physician-patient interviews, female patients exhibited cooperative interruptions more frequently than male patients [54]. Whether this might be the case for the female students in our study and account for the higher frequency of interruptions by female students requires further investigation. In general, female as well as male students in our study interrupted patients less frequently – except for that patient in case 3 – than primary care physicians who interrupted their patients on average after 12 s [44].

The medical students in our study show a decline of empathy during their undergraduate medical education [55]. Unfortunately, this is in line with observations of other groups in undergraduate [56] and postgraduate [57] medical students. As potential reasons for the decline of empathy, the hidden curriculum [57] as well as a lack of role models, high learning-volume, time pressure, hierarchy, cynicism, bureaucracy, and an atrophy of idealism during students’ socialization are given [56]. Positive role models and communication skills trainings with continuous student supervision with reflections and constructive feedback, which has been shown to have a positive influence on students’ performance, might help to prevent the decrease of empathy [55].

### Strengths and weaknesses of this study

A strength of our study is the special format of a validated competency based assessment [37] with video material of 150 student-patient encounters. One weakness of this project is that only the CARE questionnaire is a validated instrument while the observation forms for signs of verbal and non-verbal communication were designed using aspects from the literature. Another weakness of our study is the large difference in numbers between male and female participants even though it resembles roughly the actual percentage of 60% female medical students in our cohorts. Another strength of this project is the external rating of the patient interviews with the CARE questionnaire, which is independent of the personal perception of empathy by the simulated patients. An additional weakness is the fact that the participant-patient encounters were only filmed with one camera, which does not allow for a very differentiated analysis of the facial mimic of participant and patient. Furthermore, the camera was visible and could have influenced the participants and the standardized patients in their reactions. However, a strength is that a similar format of videotaping is used in our communication course, which allows differentiated video feedback to the participants.

## Conclusions

In conclusion, undergraduate medical students display differentiated communication behaviour with respect to verbal and non-verbal aspects and empathy in a competency-based assessment. While their verbal communication correlated significantly with their non-verbal communication but not with their empathy, their empathy correlated significantly with their non-verbal communication. Female students interrupted the simulated patients earlier than male students but showed in several cases significantly more signs of non-verbal communication. Since verbal and non-verbal aspects of communication are known to have an important impact on the physician-patient-encounter, the differences in communicatory aspects measured in our study suggest explicit teaching of verbal and non-verbal aspects of communication in communication classes during undergraduate training. Assessing different aspects of communication under simulated circumstances could be an important means for giving feedback to the students.

## Abbreviations

CARE: Consultation and Relational Empathy Questionnaire
UHTRUST: Utrecht Hamburg Trainee Responsibility for Unfamiliar Situations Test
